# The immune response to SARS-CoV-2 in people with HIV

**DOI:** 10.1038/s41423-023-01087-w

**Published:** 2023-10-11

**Authors:** Maxine A. Höft, Wendy A. Burgers, Catherine Riou

**Affiliations:** 1 Institute of Infectious Disease and Molecular Medicine, University of Cape Town, Cape Town, South Africa; 2 Division of Medical Virology, Department of Pathology, University of Cape Town, Cape Town, South Africa; 3 Wellcome Centre for Infectious Diseases Research in Africa, University of Cape Town, Cape Town, South Africa

**Keywords:** SARS-CoV-2, HIV, Immune dysfunction, Vaccine efficacy, Prolonged infection, Immunogenicity, Viral infection, Cellular immunity, Humoral immunity

## Abstract

This review examines the intersection of the HIV and SARS-CoV-2 pandemics. People with HIV (PWH) are a heterogeneous group that differ in their degree of immune suppression, immune reconstitution, and viral control. While COVID-19 in those with well-controlled HIV infection poses no greater risk than that for HIV-uninfected individuals, people with advanced HIV disease are more vulnerable to poor COVID-19 outcomes. COVID-19 vaccines are effective and well tolerated in the majority of PWH, though reduced vaccine efficacy, breakthrough infections and faster waning of vaccine effectiveness have been demonstrated in PWH. This is likely a result of suboptimal humoral and cellular immune responses after vaccination. People with advanced HIV may also experience prolonged infection that may give rise to new epidemiologically significant variants, but initiation or resumption of antiretroviral therapy (ART) can effectively clear persistent infection. COVID-19 vaccine guidelines reflect these increased risks and recommend prioritization for vaccination and additional booster doses for PWH who are moderately to severely immunocompromised. We recommend continued research and monitoring of PWH with SARS-CoV-2 infection, especially in areas with a high HIV burden.

## Introduction

Given the heightened vulnerability of people with HIV (PWH) to respiratory infections such as influenza and pneumococcal pneumonia [1, 2], the emergence and rapid spread of the novel coronavirus SARS-CoV-2 causing COVID-19 in December 2019 raised major concerns regarding its impact on PWH. In response, the Centers for Disease Control and Prevention (CDC) highlighted in March 2020 that individuals living with HIV might be at increased risk of severe health complications from COVID-19 compared to the general population [3]. However, it is crucial to acknowledge that PWH constitute a remarkably heterogeneous population in terms of immune competence. This immunological diversity must be considered when evaluating the intersection of the HIV and COVID-19 pandemics. Notably, there are clear distinctions between untreated PWH and those on antiretroviral therapy (ART). ART has revolutionized the management of HIV by effectively controlling viral replication and restoring immune function. Nevertheless, the extent and speed of immune restoration can vary significantly among individuals, and it is estimated that despite persistent virological suppression, normalization of CD4+ T-cell counts does not occur in up to 30% of patients [4, 5]. These individuals experience incomplete immune recovery, which is characterized by persistent systemic inflammation and lingering immune impairment. Additionally, concurrent infections and/or coexisting medical conditions in PWH further contribute to the observed heterogeneity. Hence, the persistent immunopathology often seen in chronic HIV infection might hinder immune responses to SARS-CoV-2 infection and vaccine effectiveness. Indeed, suboptimal immune responses and reduced duration of protection to several vaccines have been reported in PWH [6, 7]. Considering the current estimate of 38.4 million [33.9–43.8 million] PWH worldwide, with approximately two-thirds residing in Africa [8], a systematic evaluation of the immunological interplay between HIV and SARS-CoV-2 infection is warranted.

This review aims to cover key clinical, virological and immunological aspects of SARS-CoV-2 and HIV coinfection, including COVID-19 severity and outcome, immune responses to SARS-CoV-2, variant emergence and vaccine responsiveness. Understanding these interactions is essential for developing targeted strategies to protect and improve the health of PWH.

## Real world data: incidence, disease severity and mortality in PWH

### Incidence

Several studies have examined the incidence of SARS-CoV-2 infection in diverse cohorts to determine whether PWH are at higher risk. During the early stage of the COVID-19 pandemic, inconsistent data emerged, reporting the following: i) a higher incidence of SARS-CoV-2 infection in PWH from a San Francisco cohort, with a 4.5% positivity rate compared to 3.5% among tested people without HIV [9]; ii) a lower incidence of infection in PWH compared to the general population in two large Spanish cohorts [10, 11]; and iii) a comparable incidence between PWH and HIV-uninfected persons [12–14]. These conflicting results might be attributed to the heterogeneity of the cohorts studied. For instance, in the San Francisco cohort, 56% of the PWH included were HIV-1 viremic, and 45.5% faced adverse social determinants of health, such as marginal housing or homelessness [9]. In contrast, the majority of PWH included in the Spanish cohorts were virally suppressed and reported strong adherence to social distancing measures. In a recent systematic review of 32 studies published from December 2019 to December 2021 and involving approximately 71.8 million samples (with 1.11% representing PWH), the overall risk of SARS-CoV-2 infection was found to be comparable between PWH and HIV-uninfected individuals. However, significant variation between the studies was reported [15]. Overall, most studies do not support the notion that HIV infection itself affects susceptibility to SARS-CoV-2 infection. Instead, the incidence of SARS-CoV-2 infection among PWH is most likely influenced by social inequalities and health status, which disproportionately impact this vulnerable population [16].

### Disease severity and clinical outcome

The hallmark of HIV infection is a gradual reduction in absolute CD4+ T-cell numbers, leading to compromised cellular immunity and increased susceptibility to opportunistic infections [17]. Even with effective ART, PWH may experience persistent systemic immune activation and inflammation [18]. Therefore, when the COVID-19 pandemic emerged, it was anticipated that PWH might have increased risk of severe disease and unfavorable outcomes.

Numerous studies have repeatedly demonstrated that the crude mortality rate of COVID-19 is higher among PWH than among those not infected by HIV (summarized in [19]). However, it is crucial to recognize the immunological diversity among PWH when assessing whether HIV coinfection is an independent risk factor for unfavorable COVID-19 outcomes. Accumulating evidence indicates that patients with uncontrolled HIV infection (i.e., CD4 count <200 cells/mm^3^ and/or detectable HIV viral load) are at elevated risk of severe disease and mortality [20–24]. For example, the results from a large American study using US National COVID Cohort Collaborative data showed that among PWH, individuals with a CD4 count lower than 200 cells/mm^3^ had increased odds ratios (adjusted for demographics, lifestyle factors, and comorbidities) of 1.51, 2.73, and 3.1 for severe disease, hospitalization, and death, respectively. Similarly, Kassanjee et al. reported a strong association between COVID-19-related mortality and suboptimal HIV control in a low-income, high-HIV-burden setting, even after adjusting for demographic characteristics, comorbidities, admission pressure, location, and time period [23]. In some studies, a low CD4 count (< 200 cells/mm^3^) appeared to be a critical contributing factor to adverse COVID-19 outcomes, outweighing viral suppression as a significant contributor [20, 25].

Conversely, examining the impact of HIV on COVID-19 clinical outcomes in PWH on effective ART (i.e., virally suppressed with restored CD4 counts) has yielded variable findings. Some studies performed in the USA and Europe reported that HIV alone was not an independent risk factor for COVID-19-related death [26–29]. Conversely, others showed that although use of ART (and viral load suppression) was associated with reduced risk of poor outcomes compared to untreated individuals, PWH on ART still had significantly higher risk of death compared to HIV-negative people [30, 31]. These discrepancies highlight the complexity of the interplay between SARS-CoV-2 and HIV.

With the widespread availability of ART, HIV has transformed from a progressive, life-threatening illness to a chronic, manageable condition. Regardless, long-term use of ART has led to a shift in the spectrum of HIV-associated diseases. While opportunistic infections were once the primary concern, PWH on ART now face increased risk of noncommunicable diseases, such as hypertension, cardiovascular disease, and diabetes [32–35]. Thus, PWH are more likely than the general population to have risk factors strongly associated with adverse COVID-19 outcomes [36], and caution needs to be exercised before inferring a potential independent effect of HIV infection on COVID-19, as numerous confounders (age, sex, comorbidities, care capacity, social factors) may lead to spurious relationships between HIV- and COVID-19-associated morbidity and mortality.

It is worth mentioning that at the onset of the pandemic, it was speculated that some antiviral drugs commonly included in ART regimens might act as prophylactic treatment for SARS-CoV-2 infection owing to their in vitro effects on SARS-CoV-2 replication. For example, tenofovir, a nucleotide analog, docks in the active site of the SARS-CoV-2 protease [37]. A multicenter Spanish cohort study of ~77,000 PWH reported that a tenofovir-based ART regimen was associated with lower rates of SARS-CoV-2 infection and hospitalization [11]. However, conflicting results have been reported in different cohort studies [12]. Lopinavir, a protease inhibitor, has been shown to inhibit replication of SARS-CoV and SARS-CoV-2 in vitro [38, 39]. Nevertheless, despite its potential antiviral activity, clinical trials showed that a lopinavir-ritonavir regimen did not improve clinical outcome or mortality in COVID-19 patients [40, 41]. Overall, to date, there is no strong evidence that ART has a direct effect on SARS-CoV-2 replication in vivo.

## Immune response to SARS-CoV-2 infection in PWH

**Fig. 1:**
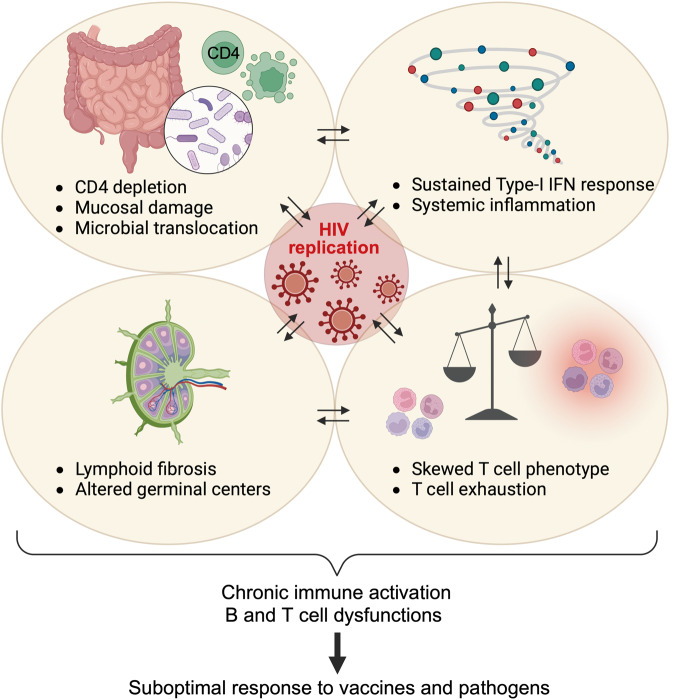
HIV-associated immune dysregulation. In the early stages of HIV infection, a rapid burst of viral replication leads to rapid depletion of mucosal CD4+ T cells. Slower and progressive depletion of CD4+ T cells in peripheral tissues and blood ensues. HIV has a major impact on gut-associated lymphoid tissue and disrupts intestinal epithelial integrity, resulting in microbial translocation and onset of chronic immune activation and inflammation. This persistent immune activation further contributes to progressive depletion of CD4+ T cells, leading to alterations in T-cell phenotype and promotion of T-cell exhaustion. HIV infection also damages the fibroblastic reticular cell network within lymphoid tissues (LTs), affecting LT architecture and subsequently impacting germinal center reactions. The cumulative effect of HIV on the immune system compromises the capacity of the host to effectively coordinate immune responses against other pathogens or respond optimally to vaccines

Although PWH represent a particularly vulnerable population due to HIV-associated impairments affecting both the innate and adaptative arms of the immune system (Fig. 1), high-quality studies comparing the magnitude and quality of the immune response following natural SARS-CoV-2 infection between PWH and those without HIV are still limited.

### Innate immunity

Innate immune cells, such as macrophages, monocytes, dendritic cells, neutrophils, and innate lymphoid cells, constitute the initial line of defense against pathogens. SARS-CoV-2 triggers recognition through distinct sets of pattern recognition receptors (PRRs), including Toll-like receptors (TLRs), retinoic acid-inducible gene-I (RIG-I)-like receptors (RLRs) and nucleotide-binding oligomerization domain (NOD)-like receptors (NLRs). Upon recognition, signal transduction occurs through downstream transcription regulators, called interferon regulatory factors (IRFs), to elicit type I and III interferon production and proinflammatory cytokines and chemokines [42]. Despite accumulating evidence showing that SARS-CoV-2 is equipped with evasion mechanisms to circumvent cellular detection and limit IFN responses [43], generation of an IFN response remains critical to control SARS-CoV-2 infection. Notably, critical and fatal cases of COVID-19 have been associated with delayed and dysregulated type I IFN (IFN-α and IFN-β) production [44]. Furthermore, inborn errors of type I IFN immunity and the presence of autoantibodies targeting type I IFNs are risk factors for life-threatening COVID-19 [45, 46]. During acute HIV infection, replication of the virus leads to activation of the innate immune system, generating an inflammatory environment associated with induction of type I IFN. Moreover, persistent type I IFN signaling, likely fueled by continued PRR triggering by replicating HIV, has been observed during the chronic phase of HIV infection [47]. This sustained response has a detrimental effect, leading to systemic immune activation, increased cell turnover and desensitization to TLR signals [48]. It could be speculated that HIV-associated chronic inflammation imprints innate cells for attenuated responsiveness, which would subsequently limit optimal responses to secondary infections such as with SARS-CoV-2. To date, it is still unknown whether HIV infection has an impact on the innate response to SARS-CoV-2 infection.

### T-cell-mediated immunity

SARS-CoV-2 infection typically triggers robust cell-mediated immunity, with SARS-CoV-2-specific CD4+ T cells outnumbering CD8+ T-cell responses [49]. Moreover, it has been observed that early generation of virus-specific T-cell responses is associated with milder disease and faster viral clearance [50]. Several studies comparing the T-cell response induced by SARS-CoV-2 infection in people with or without HIV have found no significant difference in the frequency of SARS-CoV-2-specific CD4+ T cells, either during the acute phase of infection [51] or in the convalescent state [52–54]. It is worth mentioning that these studies included mostly participants with well-controlled HIV infection. However, it has been repeatedly observed that in PWH, the magnitude of SARS-CoV-2-specific CD4+ T-cell responses is positively associated with the CD4/CD8 ratio [52] or absolute CD4 count [51, 55, 56]. This association between immune competence and the ability of the host to mount a T-cell response toward SARS-CoV-2 is further emphasized by the fact that some patients with advanced HIV have undetectable SARS-CoV-2 T-cell responses despite confirmed infection [56, 57]. Overall, this indicates that severe lymphopenia in untreated HIV patients or incomplete restoration of the CD4 compartment in ART-treated patients can hinder their ability to mount an optimal T-cell response toward SARS-CoV-2.

In addition to its quantitative effects on CD4+ T cells, HIV infection leads to qualitative defects. HIV-associated systemic immune activation may alter T-cell properties, shifting the memory profile of T cells toward an effector phenotype and increasing expression of exhaustion/inhibitory and activation markers (such as CD57, PD-1 and HLA-DR) on memory subsets [58]. During chronic HIV infection, these changes have been associated with disruption of cellular metabolic activity (e.g., cellular respiration) in most immune cells (T cells, B cells and NK cells), leading to impaired cell functionality [59]. Although ART has been shown to reverse such defects, accumulating evidence indicates that only partial restoration commonly occurs [59, 60]. Consequently, it is conceivable that SARS-CoV-2-specific T cells exhibit altered functionality in PWH. However, when assessing the ability of SARS-CoV-2-specific CD4+ T cells to produce classical Th1 cytokines (IFN-γ, IL-2 and TNF-α), no skewing in the polyfunctional profile was observed in virally suppressed PWH compared to HIV-uninfected individuals [51, 52]. One study, however, reported that in the covalescent phase, SARS-CoV-2-specific CD4+ T cells in PWH were characterized by elevated expression of PD-1 compared to HIV-uninfected individuals [53]. It remains to be determined whether such attributes affect memory recall responses upon pathogen re-encounter.

As the current literature assessing the impact of HIV infection (treated or not) on SARS-CoV-2 T-cell responses is still limited, several questions remain unanswered. To date, T-cell responses have been almost exclusively analyzed in blood. Only 2% of total lymphocytes are found in the circulation, with the remainder distributed throughout the body, especially in lymphoid organs such as lymph nodes and gut-associated lymphoid tissue (GALT). HIV infection is known to damage the lymphoid tissue (LT) fibroblastic reticular cell network, altering the architecture of the LT, which subsequently impacts the germinal center reaction [61–63]. These architectural abnormalities are not readily reversed with effective ART, especially with late initiation of treatment [64, 65]. Hence, due to the long-lasting impact of HIV infection on lymph node and gut architecture, it remains to be defined whether tissue-resident responses to SARS-CoV-2 are altered in PWH. Moreover, while accumulating evidence shows broad and long-term SARS-CoV-2-specific T-cell responses in recovered COVID-19 patients with an estimated half-life of 200 days [49], longitudinal analyses assessing the long-term durability of the T-cell response in PWH are lacking.

### Humoral immunity

Development of a robust neutralizing antibody response against SARS-CoV-2 is a crucial factor in providing protection against COVID-19 and reinfection [66]. Studies comparing the magnitude and persistence of humoral responses following SARS-CoV-2 infection between PWH and those without HIV infection have yielded mixed results. On the one hand, Snyman et al. reported similar titers and times to peak response of SARS-CoV-2-specific IgM and IgG during acute SARS-CoV-2 infection in both PWH and HIV-uninfected individuals [67]. Furthermore, studies involving convalescent COVID-19 patients have demonstrated comparable SARS-CoV-2-specific IgG titers and neutralization potency between PWH and HIV-uninfected individuals [52, 67–69], suggesting a similar durability of the SARS-CoV-2-induced humoral response, irrespective of HIV status. However, most participants included in these studies had well-controlled HIV infection. In contrast, two studies conducted in the USA and in South Africa reported reduced neutralization responses to SARS-CoV-2 and Spike-specific IgG in PWH compared to HIV-uninfected individuals, with the greatest reduction observed in PWH with uncontrolled HIV viremia and/or CD4 count <200 cells/mm^3^ [70–72]. It is important to mention that in all these studies, regardless of the type of assay used or group studied, the magnitude of the antibody response against SARS-CoV-2 was highly variable, ranging up to 1000-fold between individuals. In addition to inherent person-to-person immune diversity, this heterogeneity might be partly explained by differences in host immune competence, and several HIV-associated impairments may account for the suboptimal antibody response to SARS-CoV-2 observed in some PWH.

T follicular helper (Tfh) cells are crucial for orchestrating functional humoral immunity by supporting B-cell activation and antibody generation. Indeed, pathogen-specific CXCR5-expressing Tfh cells relocate in the germinal centers (GCs) of secondary lymphoid organs and provide help to B cells, facilitating class switch recombination, somatic hypermutation and generation of long-lived antibody-secreting B cells [73]. Hence, the quality of the B-cell response following SARS-CoV-2 infection determines the duration and breadth of protective immunity [74]. Several studies have shown that SARS-CoV-2-specific Tfh cell responses occur following SARS-CoV-2 infection, with a correlation between the frequency of circulating Tfh cells and neutralizing antibody titers [75–77]. Notably, severe or fatal COVID-19 cases have been associated with a drastic reduction in GC formation, which is most likely linked to defective Bcl-6+ Tfh cell generation [78]. Tfh dysregulation during HIV infection has been well documented [63]. Tfh cells appear to be highly susceptible to HIV infection and may play an important role as a cellular reservoir for HIV persistence [79]. Moreover, despite the observed expansion of Tfh cells during acute and chronic HIV infection, GC Tfh cells from HIV patients exhibit a reduction in key signaling mediators (IL-6R and Stat-3) and expression of genes implicated in costimulation (Ox40, CD40L and ICOS), potentially compromising high-affinity B-cell maturation and development of long-lived memory B cells [80–82]. While the specific impact of HIV infection on the SARS-CoV-2-specific Tfh response is unknown, alterations in the B-cell response have been well documented in PWH. Krause et al. reported that memory B cells in PWH display an altered phenotype, with a reduced proportion of IgD- CD27+ class switched memory B cells, elevated IgD-CD27- double-negative B cells and reduced expression of CXCR5, a marker associated with B-cell migration to GCs [83]. These modifications shift the B-cell response away from GC maturation, favoring an extrafollicular pathway characterized by reduced affinity maturation and suboptimal development of long-lived memory B cells, which can affect antibody recall responses. Such HIV-associated alterations of B-cell maturation may explain findings by Hwa et al. showing that the antibody response in the second wave of SARS-CoV-2 infections was affected by HIV status, with PWH mounting less effective IgG responses to the Beta variant [72]. Of note, comparable alterations in the phenotype of SARS-CoV-2-specific B cells in PWH compared to heathy controls have also been observed after SARS-CoV-2 mRNA vaccination [84]

**Fig. 2:**
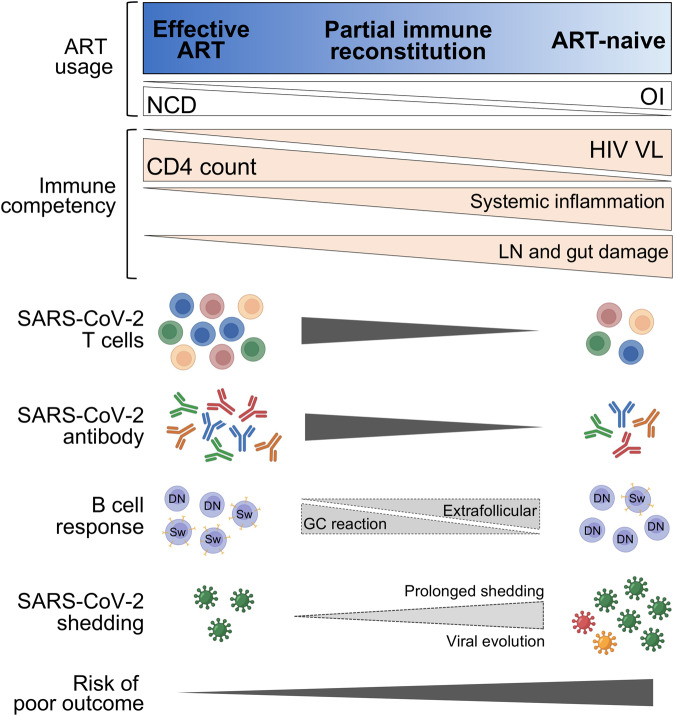
Immune responses to SARS-CoV-2 infection in PWH depend on their immune competency. PWH with well-controlled HIV infection (effective ART) show a humoral and T-cell response comparable to HIV-uninfected individuals, as characterized by strong T-cell and antibody response targeting multiple regions across the SARS-CoV-2 viral genome. Conversely, in ART-naïve individuals with low CD4 counts, generation of T-cell responses is suboptimal and of reduced frequency. Antibody responses are also compromised, likely due to HIV-induced changes in the architecture of lymph nodes, skewing the B-cell response toward an extrafollicular pathway. The suboptimal nature of the immune response in this context may lead to delayed clearance of SARS-CoV-2, creating an environment conducive to viral evolution. ART antiretroviral therapy, NCD noncommunicable diseases, OI opportunistic infections, LN lymph node, GC germinal center, Sw Switched memory B cells (IgD-CD27+), DN IgD-CD27- double negative B cells

Overall, according to our current knowledge, the immune response to SARS-CoV-2 in PWH with well-controlled infection appears comparable to the responses in those without HIV. However, in PWH with low CD4 counts and/or uncontrolled HIV viremia, a suboptimal cellular and humoral immune response is mounted, potentially heightening their risk of severe disease and reinfection (Fig. 2).

## Prolonged shedding and viral evolution in PWH

The COVID-19 pandemic has been characterized by emergence of variants of concern (VOCs) with greater transmissibility and increasing ability to evade neutralizing antibody responses induced by vaccination or infection, reducing the ability of vaccines to provide sterilizing immunity [85]. One leading hypothesis for emergence of the Omicron variant, which represented a major evolutionary shift from previous VOCs, is that the virus evolved a hypermutated Spike protein over a lengthy period in a single host until these mutations conferred the ability to transmit efficiently [86]. Sustained viral transmission in immunologically experienced populations enables viral evolution and acquisition of adaptations. Acute infections typically last one to two weeks, which is not a sufficient duration for in vivo evolution before onward transmission. Prolonged SARS-CoV-2 infection, however, would provide an extended period for within-host evolution, with mutations accumulating in the viral genome under immune selective pressure from the host [87].

Prolonged infection in immunocompromised patients was documented early in the pandemic, including patients with cancers, organ transplants, and primary immunodeficiencies as well as those treated with immunosuppressive medications [88–91]. Protracted infection has now been described in at least 21 people with advanced HIV [92–110]. Overall, data thus far suggest that severe immune dysfunction, rather than any properties of HIV itself, is the cause of prolonged infection. Indeed, advanced HIV infection is characterized by low CD4+ T-cell counts, and the ensuing immune deficits in adaptive immunity may lead to an inability to eliminate SARS-CoV-2. A murine study demonstrated that immunodeficient mice that lacked mature B and T cells developed chronic infection with SARS-CoV-2, with high levels of infectious virus persisting in the respiratory tract [111]. Consistent with this finding, a nonhuman primate study found delayed viral clearance when CD4+ and CD8+ T cells were depleted prior to viral challenge [112]. As discussed previously, Tan et al. reported that early functional T-cell responses are essential for clearing SARS-CoV-2 infection swiftly [50]. Thus, during advanced HIV infection, depletion or dysfunction of the CD4+ T cells that provide help to B cells for development of functional antibody responses and to CD8+ T cells for development of memory responses may result in a delayed ability to clear SARS-CoV-2 infection and sustain ongoing viral replication.

Although these studies of prolonged infection in PWH are limited to case reports or case series, they nonetheless provide important insights into the behavior of SARS-CoV-2 in the context of HIV coinfection. The median duration of viral shedding in these studies was 109 days (range, 35–270) [92–110]. A notable common feature was that patients having been newly diagnosed with HIV infection or poorly adherent to ART showed ultralow CD4 counts (<50 cells/mm^3^) and HIV viral loads exceeding 100,000 mRNA copies/ml. Only two studies have described prolonged infection in individuals with normalized CD4 counts and undetectable viral loads [95, 101], though the mechanism of SARS-CoV-2 persistence in these patients was not explored. Larger cohort studies support a longer duration of SARS-CoV-2 replication in advanced or uncontrolled HIV infection. A hospitalized cohort of PWH from South Africa demonstrated significantly longer shedding of high levels of SARS-CoV-2 (determined by the Ct value) in PWH with CD4 counts <200 cells/mm^3^ or with a detectable HIV viral load (median 26 days) compared to HIV-uninfected persons and those with CD4 counts >200 or virally suppressed, with virus clearance after a median of 7 days [113]. This report was consistent with findings from a community cohort from South Africa [114], as also supported by a cohort study of hospitalized Chinese PWH who had a median duration of viral shedding of 30 days [13].

The most remarkable observation from these studies of prolonged SARS-CoV-2 infection in PWH is that the virus accumulated multiple mutations across the genome, including in the N-terminal and RBD regions of Spike [94, 95, 97, 100, 104, 105, 107]. Extensively mutated Spike sequences share key amino acid changes that were found or later arose in VOCs, including mutations associated with neutralization escape. Cele et al. directly demonstrated the neutralization resistance that developed in evolving viral isolates toward the patient’s own sera and sera from convalescent and vaccinated individuals [94]. By testing early sera and later viral variants, and vice versa, this group elegantly demonstrated sequential evolution of neutralization escape over 6 months of persistent SARS-CoV-2 infection. Some studies have shown that immune-evasive mutations in viral variants accumulate within a few weeks, highlighting that intrahost evolution can be rapid [94, 97]. Collectively, these studies demonstrate that prolonged infections may give rise, independently and repeatedly, to mutations present in VOCs that confer resistance to neutralizing antibodies.

Some patients with prolonged infection had significant coinfections and comorbidities, owing to advanced HIV disease [96, 99, 106, 107], but there were also several instances of asymptomatic carriage of SARS-CoV-2 for extended periods [94, 102]. Furthermore, based on viral culture and cell fusion experiments, it was reported that viral isolates remained infectious [94, 108, 115], underscoring the risk of potentially successful transmission of variants with novel mutations. Prolonged infection in PWH has been described for all major variants, including the ancestral strain [94], Alpha [102, 106], Beta [100], Delta [104] and Omicron [92]. In fact, several of these cases were recognized through routine genomic sequencing that identified a VOC no longer in circulation. It is worth noting that long-term infections may also increase risk of superinfection, which in turn amplifies the possibility of viral recombination [116], accelerating emergence of new viral properties. The successful and widespread XBB lineage of Omicron represents recombination between two BA.2 lineages [117]. Given these considerations, targeted genomic surveillance may be warranted in PWH.

A striking feature of several studies is that initiation or resumption of ART in these patients facilitates SARS-CoV-2 clearance, highlighting a critical mechanism for reducing risk of long-term infection [94, 97, 100, 110]. In some cases, relatively rapid resolution of infection occurred, indicating that full immune reconstitution is not necessary for SARS-CoV-2 clearance. It is thus clear that controlling HIV may contribute to controlling COVID-19. In addition to HIV treatment, modeling suggests that better antiviral treatments for SARS-CoV-2 infection for this group can substantially lower the probability of novel variants emerging [118].

It is important to note that none of the evolved viruses described in the case studies of PWH were shown to have successfully been transmitted to others; rather, they suggest potential pathways for emergence of new variants. A risk of highlighting the importance of PWH in contributing new COVID-19 variants is further stigma for this group. It is worth considering that PWH are among a larger group of immunocompromised patients who may not have adequate immunity to SARS-CoV-2, even after vaccination. While treatment options for immunocompromised patients with prolonged infection are generally limited, existing antivirals and monoclonal antibodies for SARS-CoV-2 are unavailable in most of the parts of the world where the majority of PWH reside. It is thus imperative that improved care and access to the best available treatments for SARS-CoV-2 be prioritized, in addition to HIV care [119].

## COVID-19 vaccine efficacy in PWH

A range of highly effective vaccines have been developed and deployed rapidly to prevent COVID-19 [120–126]. Vaccine efficacy is measured against infection (symptomatic or asymptomatic), hospitalization (severe and critical disease), and death. A range of factors influence vaccine efficacy, including vaccine type, time since vaccination, circulating variant and immune status of the vaccinee. While the vaccines in widespread use perform well in preventing infection against the ancestral strain of SARS-CoV-2, emergence of immune-evasive variants lower vaccine efficacy against infection, and breakthrough infections are now common [124, 127, 128]. Nonetheless, the protective efficacy of vaccines against severe COVID-19 has remained high [128, 129].

PWH are underrepresented in the large clinical efficacy trials undertaken to test the major vaccines in widespread use. While PWH on stable ART and with well-controlled viremia were included in some trials, those with advanced HIV were excluded. PWH represented only 1,557 (1%) of a total number of 149,063 participants for phase 2/3 trials testing mRNA-1273/Moderna, BNT162b2/Pfizer-BioNTech, ChAdOx1/AstraZeneca, NVX-2373/Novavax and Ad26.COV2.S/Janssen vaccine efficacy [121–123, 125, 126], with other major vaccine trials excluding or not reporting numbers of PWH. One result that emerged from the Novavax trial in South Africa was that vaccine efficacy was demonstrably lower in those seronegative for SARS-CoV-2 when PWH were included in the analyses (60.1% for HIV-uninfected individuals *vs*. 49.4% when PWH were included). However, low numbers of PWH generally preclude robust subgroup analysis to detect differences in vaccine efficacy, rendering the question of vaccine efficacy in this group unresolved.

In addition to randomized controlled trials testing efficacy, real-world effectiveness studies, including test-negative case‒control designs or retrospective cohort studies, have reported vaccine performance in PWH. While observational, these studies featured longer follow-up and evaluated vaccine performance in a more heterogeneous group of PWH at the population level, and all were performed pre-Omicron emergence. To date, the largest study of PWH was a phase 3 open-label implementation trial of a single dose of Ad26.COV2.S/Janssen vaccine in 477,102 health care workers in South Africa, of whom 8.3% were PWH, mostly women [130]. Similar effectiveness was demonstrated for health care workers with HIV compared to HIV-uninfected health care workers for hospital admissions, including critical care. A higher number of COVID-19 deaths in vaccinated PWH compared to the HIV-uninfected group was reported, though total deaths were low because the vaccine maintained good efficacy against fatal COVID-19; therefore, these analyses are limited. Chambers et al. found similar vaccine efficacy against symptomatic disease and severe outcomes in PWH in Canada who were seeking care and generally healthy with viral suppression [131]. Another Canadian study reported comparable vaccine effectiveness in PWH compared to a matched HIV-uninfected group at the peak timepoint measured (2 weeks to 4 months after two vaccine doses) [132]. However, waning protection appeared to occur more rapidly in PWH, with vaccine effectiveness of 58.2% for laboratory-confirmed infection in PWH compared to 84.2% in HIV-uninfected controls at 4–6 months after vaccination. The adenoviral-vectored Sputnik vaccine was demonstrated to protect PWH with CD4 counts >350 cells/mm^3^ with high effectiveness, but there was a trend toward lower effectiveness in those with CD4 counts <350 cells/mm^3^ [133]. These latter reports consisted mostly of men with HIV, highlighting the importance of performing these studies in different parts of the world where HIV affects populations differently and where the standard of care may differ.

An increased number of breakthrough infections in PWH has been reported, indicating higher risk of infection, even after vaccination. Coburn et al. reported 28% higher risk of infection after vaccination in PWH after 9 months [134]. Higher CD4 counts (>500 cells/mm^3^ *vs*. <200 cells/mm^3^) were associated with a reduced risk of breakthrough infection. An important observation in this study was that an additional (third) vaccine dose decreased risk of breakthrough infections in PWH. In a large study of immunocompromised patients, Sun et al. also demonstrated elevated risk of breakthrough infection in PWH [135].

In light of these findings, vaccination guidelines have sought to prioritize PWH with advanced HIV or uncontrolled disease for vaccination and provide them with additional booster doses. The updated ‘*WHO SAGE roadmap on uses of COVID-19 vaccines in the context of OMICRON and substantial population immunity’* (30 March 2023) places PWH in the ‘high priority’ group for vaccination if they have a CD4 count <200 cells/mm^3^, unsuppressed/detectable viral load or an opportunistic infection [136]. These individuals are grouped together with those with medium to high immunocompromising conditions, including those receiving immunosuppressive medications. These guidelines are consistent with recommendations from several regions [136, 137].

## Immune response to COVID-19 vaccines in PWH

Given the potential for adverse outcomes after SARS-CoV-2 infection, an important consideration is whether optimal immune responses after COVID-19 vaccination are mounted in PWH. There is evidence that some vaccines elicit suboptimal responses in PWH as a result of persisting immune dysfunction, exhaustion and immune senescence, even after CD4 reconstitution and HIV suppression by ART [138]. For example, impaired humoral response to influenza vaccination has been well documented in PWH [139].

Given the potential of compromised vaccine responses in PWH, numerous studies have characterized the immunogenicity of COVID-19 vaccines in this group. These studies encompass a range of vaccine modalities, including mRNA vaccines (mRNA-1273/Moderna or BNT162b2/Pfizer-BioNTech) [140–160], viral vectored vaccines (ChAdOx1/AstraZeneca or Ad26.COV2.S/Janssen) [71, 127, 151, 161, 162], inactivated vaccines (BBIBP-CorV/Sinopharm or CoronaVac/Sinovac BioTech) [163–171], and a protein subunit vaccine (NVX-2373/Novavax) [172]. Moreover, some studies have explored heterologous vaccine regimen immunogenicity in PWH [173, 174]. Most of these studies measured humoral responses to COVID-19 vaccines in PWH shortly after vaccination, and a more limited number investigated T-cell responses after vaccination [140, 144, 148, 161, 166, 168].

### Humoral responses in PWH after vaccination

Humoral responses have been assessed as Spike IgG seroconversion, Spike-binding antibody responses, and Spike neutralization titers.

#### Seroconversion

The seroconversion rate postvaccination is a marker of successful generation of vaccine Spike-specific antibodies. Twelve studies reporting seroconversion 2 weeks to 3 months after mRNA vaccination showed no differences between PWH and controls [141–148, 150, 157, 173, 175]. Of note, the majority of PWH in these studies were receiving effective ART and had CD4 counts well above 200 cells/mm^3^, with suppressed HIV viral loads (<50 mRNA copies/ml). Antinori et al. reported that seroconversion rates were comparable to controls in PWH with CD4 counts in the strata >500 cells/mm^3^ and 200–500 cells/mm^3^ but that those with CD4 counts <200 cells/mm^3^ exhibited lower seroconversion rates compared to controls [140]. Similarly, following adenoviral-vectored COVID-19 vaccination, no differences were observed in the seroconversion rate of PWH (CD4 counts >350 cells/mm^3^, HIV viral loads <50 mRNA copies/ml) compared to controls [161]. However, six studies have reported a significantly lower seroconversion rate in PWH compared to controls after receiving two doses of inactivated SARS-CoV-2 vaccine, irrespective of CD4 count [163–167, 170], with only a single dissenting study reporting comparable seroconversion rates after inactivated vaccine in PWH with CD4 count >200 cells/mm^3^ and controls [169]. A meta-analysis that examined multiple risk factors across 23 different studies confirmed these findings by establishing associations between CD4 count and vaccine type with seroconversion in PWH following COVID-19 vaccination [176].

#### Binding antibodies

While many studies have shown comparable seroconversion rates after mRNA vaccination between PWH and controls, the magnitude of the antibody response differs. Some studies reported no differences in titers of binding antibodies in PWH following mRNA vaccination compared to HIV-uninfected individuals [141, 143, 144, 148], while others reported a significantly lower IgG titer in PWH, regardless of their CD4 count [140, 142]. These divergent results are not surprising, given the heterogeneity of PWH, which is supported by a few studies that stratified antibody titer by CD4 count. These studies report comparable binding antibody titers after mRNA vaccination in PWH with CD4 counts >500 cells/mm^3^ and a significant decrease in groups with lower CD4 T-cell counts, particularly those below 200 cells/mm^3^ [157, 169, 175, 177, 178]. A single study compared IgG titers following adenoviral vectored vaccine in PWH and reported no differences at 1 month post-vaccination compared to controls [161]. Consistent with the low conversion rates reported, it has been observed that PWH exhibit significantly weaker binding antibody responses after receiving an inactivated vaccine compared to control groups [163–165, 167], particularly those with CD4 <200 cells/mm^3^ [163, 167, 169].

#### Neutralizing antibody responses

Neutralizing antibodies strongly correlate with protection against SARS-CoV-2 infection [179]. The magnitude of neutralizing antibody responses reported following COVID-19 vaccination varies across studies and vaccine modalities, partially attributable to different neutralization readouts and assays. Similar SARS-CoV-2 neutralizing antibody titers were reported between PWH and controls after mRNA vaccination in several studies [143, 148, 149, 152, 180]. In contrast, two studies reported significantly lower neutralizing responses in PWH at one to three months post mRNA vaccination [147, 175]. As reported for binding antibodies, advanced immune deficiency compromises neutralizing responses, and two studies reported a significant reduction in the magnitude of SARS-CoV-2 neutralizing antibodies in PWH with CD4 counts <200 cells/mm^3^ at one month after mRNA vaccination. No such differences were detected in PWH with high CD4 counts (>500 cells/mm^3^) [140, 178].

Measurement of neutralizing titers in PWH who received an inactivated vaccine has yielded mixed results. Some studies report lower neutralizing titers in PWH compared to controls [164, 167], while Cai et al. described comparable neutralizing titers between PWH and controls [165]. Huang et al. revealed a more nuanced pattern, whereby PWH with CD4 counts exceeding 500 cells/mm³ exhibited comparable neutralizing activity to controls after inactivated SARS-CoV-2 vaccination. However, those with CD4 T-cell counts below 500 cells/mm³ exhibited a marked decrease in neutralizing activity [168]. This disparity in vaccine response among PWH may be attributed to variations in the mechanisms by which different vaccine types deliver antigens to cells or provide costimulatory signals during the initiation of an immune response. These variations might impact the capacity to elicit an effective immune response in PWH, especially for those with specific immunological deficits such as impaired antigen presentation, in advanced stages of HIV disease.

As vaccine-induced neutralizing antibodies are primed to the ancestral Spike used in first-generation vaccines, an important consideration is how well they inhibit subsequent VOCs that have emerged. When evaluating mRNA vaccine-elicited antibody neutralization against the Alpha, Beta, and Gamma variants [148], as well as adenovirus-vectored vaccine responses [181], no discernible differences between PWH and controls were observed. Finally, some studies have directly compared different vaccine modalities in head-to-head comparisons, with growing evidence that mRNA vaccines elicit the highest SARS-CoV-2-specific binding and neutralizing antibodies in both PWH and HIV-uninfected persons [154, 173, 180, 182, 183].

### T-cell responses after vaccination in PWH

There is ample evidence for the vital role of virus-specific T-cell responses, in addition to neutralizing antibodies, in limiting severe COVID-19 disease [184]. Robust spike-specific T-cell responses are generally elicited after vaccination in PWH [140, 144, 148, 161, 164, 168]. SARS-CoV-2-specific T-cell responses after vaccination vary across studies depending on the immunological status of the PWH, vaccine type, and time interval after a completed vaccination series. Across three different vaccine modalities (mRNA, viral vectored and inactivated vaccines), multiple studies have reported no significant differences in Spike-specific IFN-γ production at 2 weeks to 6 months after completion of primary vaccination [144, 148, 161, 162, 164, 166, 177]. These studies employed a range of assays to measure T-cell responses, including flow cytometry, IFN-γ ELISpot and IFN-γ release assays. Despite no significant differences in the magnitude of the T-cell response measured in these studies, the proportion of PWH who mounted a T-cell response differed depending on the immunological status of the cohort. Studies including participants with a median CD4 count ranging from 600–900 cells/mm^3^ had a comparable frequency of responders between PWH and controls [148, 161, 166]. However, a study including PWH with a median CD4 count of 254 cells/mm^3^ (ranging from 128–346 cells/mm^3^) reported that 32% had no T-cell response but that only 12% of the control group failed to mount a response. Thus, while the magnitude of T-cell responses was not significantly different between PWH and HIV-uninfected controls in these studies, the ability to mount a T-cell response was associated with a higher CD4 count. A large study by Antinori et al. stratified T-cell responses by CD4 count after mRNA vaccination (BNT162b2 or mRNA-1273) and reported no significant differences between the control group and PWH with high CD4 recovery (>500 cells/mm^3^). However, a significantly lower IFN-γ response has been reported for PWH with poor and intermediate CD4 T-cell recovery (<200 cells/mm^3^ and 200–500 cells/mm^3^, respectively) [140]. In contrast to the majority of other studies, reported T-cell response magnitudes following inactivated SARS-CoV-2 vaccination were lower in PWH than in controls, despite having CD4 counts >500 cells/mm^3^ [168].

While humoral responses appear to wane substantially by 6 months after the primary vaccination series, numerous studies have shown that T-cell responses are well maintained at 6 months after mRNA or adenoviral vector vaccination in both PWH and HIV-uninfected control groups [148, 160, 162, 177]. Interestingly, a longitudinal analysis performed by Huang et al. showed that at 2 weeks post inactivated vaccination, there was a significantly lower proportion of T-cell responders among PWH than in the HIV-uninfected group; however, T-cell responses were comparable between the two groups at 1–2 months after vaccination [168]. This might be related to a delayed T-cell response in PWH but may also be attributed to breakthrough infection. In contrast to other vaccines, the proportion of PWH mounting a Spike-specific T-cell response after administration of an inactivated SARS-CoV-2 vaccine was significantly lower than in uninfected controls [168].

Studies comparing the breadth or functional profile of T-cell responses between PWH and uninfected individuals are very limited. Woldemeskel et al. reported similar T-cell breadth following mRNA vaccination between the two groups when measuring IFN-γ production in response to 10 peptide pools spanning Spike [148]. It has also been reported, in the context of adenoviral vector (ChAdOx1) vaccination, that the proliferative potential of Spike-specific T cells in PWH is comparable to that in HIV-uninfected controls [162]. Moreover, this latter study showed that in PWH, the T cells generated upon ChAdOx1 vaccination showed robust cross-reactivity against Beta, Gamma and Delta VOCs. This is in line with data for HIV-uninfected individuals [185, 186]. However, further studies are warranted to define whether HIV infection has an impact on the polyfunctional profile of COVID-19 vaccine-induced T-cell responses.

### Booster doses

Since a number of studies have shown significantly reduced vaccine responses in PWH with low CD4 counts, vaccine boosting in this vulnerable population is an important consideration. Two studies showed that a booster (third) dose of mRNA vaccine significantly increases binding antibody and neutralization responses in PWH, including those with advanced HIV and irrespective of current CD4 count [180, 187]. Additionally, a study conducted by Sistere-Oro et al. focused on 10 PWH who were classified as immunological nonresponders, with CD4 counts <200 cells/mm^3^ despite viral suppression [188]. After two doses of mRNA vaccination, only half of the participants showed seroconversion and generated neutralizing antibodies and IFN-γ T-cell responses. Among these participants, three who had previously not responded to the initial vaccination series were offered a booster dose. All three generated Spike-specific IgG, but only one of them had a detectable T-cell response. Although this study’s sample size was small, it highlights an important subgroup of PWH who are underrepresented in many vaccine immunogenicity studies and underscores the importance of booster vaccination. For the most part, vaccine booster doses after a primary series have a positive effect on boosting antibody responses but a more limited effect on increasing T-cell memory responses. Alexandrova et al. reported that in PWH, Spike-specific T-cell response magnitudes after a booster (third) dose remained stable compared to response levels after the primary vaccination series [187]. This is consistent with a report that repeated SARS-CoV-2 exposures (either by infection or vaccination) enhance IgG responses but that T-cell memory responses plateau after two to three Spike exposures in HIV-uninfected people [189].

### Durability

It is now clearly established that anti-SARS-CoV-2 binding IgG and neutralizing antibodies, generated by either natural infection or vaccination, substantially decrease over time in the HIV-uninfected population [190, 191]. Such waning has also been reported in PWH [192]. However, it is still unknown whether the decline rate of the anti-SARS-CoV-2 humoral response is comparable between PWH and uninfected individuals. In contrast, COVID-19 vaccines lead to establishment of relatively stable Spike-specific CD4+ T-cell memory pools, which are detectable up to 6 months post vaccination [193, 194]. This persistence of the vaccine-induced T-cell response has also been shown for PWH [148]. The stability of T-cell responses beyond 6 months remains to be established.

Overall, the immune responses following COVID-19 vaccination in PWH appear to be comparable to those in people without HIV, except for individuals with CD4 counts below 200 cells/mm^3^ and detectable viremia. Consequently, PWH with advanced immunosuppression should be prioritized for booster vaccination, and increased immune surveillance is needed for this vulnerable population. Notably, viral-vectored and mRNA vaccines have superior immunogenicity in PWH compared to inactivated vaccines, aligning with the recommendations issued by the WHO in 2023 [195].

## Conclusion

COVID-19 is no longer causing severe disease and death in large numbers of people worldwide; however, global surges of infection still occur frequently, and the trajectory of viral evolution is unpredictable. Certain risk groups remain vulnerable, and vigilance is required to understand and mitigate poor outcomes in these groups. While PWH represent a heterogeneous group, those with advanced or untreated HIV may have even poorer COVID-19 outcomes. PWH in whom suboptimal responses after vaccination are mounted may also be at higher risk of infection or reinfection. This concern is reflected in global guidelines for extended COVID-19 vaccination series and making booster doses available for PWH with moderate to severe immunosuppression.

Limitations in our understanding of the interplay between COVID-19 and HIV stem from the fact that many studies are observational and include relatively small groups of PWH.

High-quality studies are lacking in certain areas, representing opportunities and priorities for ongoing research (Box 1). There is insufficient knowledge about the duration of immunity to SARS-CoV-2 after infection and/or vaccination. Long-term follow-up of PWH is required to assess the durability of humoral and cellular responses in this population. Overall, there are gaps in our understanding of several aspects of immunity to SARS-CoV-2 in PWH, such as whether innate mechanisms and Tfh cells targeting SARS-CoV-2 are deficient. It will be important to monitor whether, as for key neutralizing epitopes that are mutated during prolonged infection, escape at T-cell epitopes occurs.

Along with other immunocompromised groups, PWH who are immunosuppressed may be at risk of persistent SARS-CoV-2 infection. While long-term SARS-CoV-2 infection may not necessarily pose a greater health risk to these individuals, the potential contribution to generation of virus variants that differ in their replication capacity, infectivity and antigenicity poses a risk to populations. Of the 25.3 million people with HIV in Africa, it is estimated that 8 million are untreated or that treatment is failing [119], indicating a large number of people who need HIV care. ART is a highly effective intervention for both reducing risk of exacerbated COVID-19 outcomes and the probability of prolonged infection. However, it is precisely those individuals outside of care, who may be unaware of their HIV status, have never initiated ART or for whom treatment is failing, who are more difficult to reach. Box 2 lists several recommendations for improved HIV and COVID-19 management. Ultimately, to reduce the impact of COVID-19 on PWH, we need to reaffirm our commitment to HIV care, diagnosis of new HIV infections, ART initiation or optimization, adherence support and prevention of new HIV infections through access to the many interventions now available [196]. This will have a public health benefit not only for COVID-19 but also far more broadly for a range of diseases.

Box 1 Outstanding research questions

**Table:** 

What is the burden and risk of long-term sequelae of COVID-19 in PWH?
Are innate immune responses targeting SARS-CoV-2 compromised in PWH?
Do robust mucosal responses and tissue resident memory develop at relevant sites in PWH?
What is the magnitude and quality of vaccine-induced immune responses in PWH with low CD4 count and/or elevated HIV viral load?
How durable are immune responses after infection and/or vaccination in PWH?
What is the impact of HIV infection on SARS-CoV-2-specific follicular helper T cells after vaccination and/or infection?
What are the mechanisms of viral persistence in PWH who are immunocompetent?
Does T-cell escape emerge in prolonged SARS-CoV-2 infection in PWH?

Box 2 Recommendations

**Table:** 

Targeted genomic surveillance in immunocompromised patients, including people with advanced or uncontrolled HIV
Development of affordable and accessible treatments and treatment strategies for prolonged SARS-CoV-2 infections in PWH
Improved HIV care, including prevention and diagnosis of new infections, ART initiation or reinitiation and adherence support
Access to updated variant vaccines in low- and middle-income countries to prevent new infections in PWH
Integration of HIV primary care services and COVID-19 vaccination
